# Incident Far‐Red Photons Drive Leaf Photosynthesis Less Efficiently Than PAR Light, but Are More Effective in Promoting Growth

**DOI:** 10.1111/pce.70193

**Published:** 2025-09-16

**Authors:** Wenqing Jin, Elias Kaiser, Yingyue Peng, Yawen Gu, Ep Heuvelink, Leo F. M. Marcelis

**Affiliations:** ^1^ Horticulture and Product Physiology, Department of Plant Sciences Wageningen University and Research Wageningen Netherlands; ^2^ Priva De Lier Netherlands

**Keywords:** acclimation, Emerson enhancement effect, far‐red, light use efficiency, photosynthesis

## Abstract

Recently, far‐red light (FR) in the range 700–750 nm has been reported to have similar photosynthetic efficiency as photosynthetically active radiation (PAR, 400–700 nm), when supplied in combination with PAR. We aimed to investigate if adding FR to PAR is equally efficient in promoting photosynthesis as adding PAR, and if long‐term acclimation to FR would change the short‐term response to FR. Lettuce plants were grown in a climate chamber at two levels of PAR (200 and 400 μmol m^−2^ s^−1^, red/blue light), and at each PAR level there were also treatments with 25% of PAR or FR added. In all six treatments, response curves of leaf net photosynthesis rate (P_n_) to different intensities of PAR or PAR + FR were determined. Adding FR to PAR increased P_n_, but this was only 39%–64% of the increase seen under additional PAR, due to lower absorption of FR than PAR. Absorbed PAR and FR photons had similar photosynthetic efficiency. Leaves grown under FR showed acclimatory responses, such as reduced photosynthetic capacity and pigmentation, but the instantaneous photosynthesis response to FR was unaffected. FR had strong positive effects on growth: Partly substituting PAR by FR increased the radiation use efficiency of growth even when expressed per unit of absorbed radiation.

## Summary statement

- Absorbed far‐red photons are equally photosynthetically active as PAR light when supplied in combination with PAR light, but their absorption is lower than that of PAR light.
- Contrastingly, absorbed far‐red photons are more effective in promoting growth.

## 1 Introduction

The sun emits a large range of electromagnetic radiation, roughly half of which is used by plants to fuel photosynthesis. Far‐red (FR: 700–800 nm) light has a wavelength just above what is typically defined as photosynthetically active radiation (PAR: 400–700 nm). Adding FR to PAR may improve biomass production via at least two ways: Firstly, additional FR promotes leaf expansion, resulting in enhanced light interception and therefore may improve growth (Kalaitzoglou et al. 2019; Jin et al. 2021). Secondly, FR can improve photosynthetic efficiency. Even though FR barely contributes to photosynthesis when applied alone, together with shorter wavelengths (600–685 nm) FR powers photosynthesis efficiently and more strongly than the sum of its parts, a phenomenon named the Emerson Enhancement Effect (Emerson and Rabinowitch 1960). In the thylakoid membrane of chloroplasts, photosystem II (PSII) and photosystem I (PSI) operate in series to transfer electrons along the photosynthetic electron transport chain and carry out photochemical reactions that ultimately result in CO_2_ fixation and the production of photoassimilates. The peak wavelength for excitation of PSI is at 700 nm and that of PSII is at 680 nm (Kok 1961; Barber and Archer 2001). Therefore, applying PAR or FR alone causes imbalanced excitation between the two photosystems, resulting in a suboptimal photosynthetic efficiency (Hogewoning et al. 2012).

Several studies (Zhen and van Iersel 2017; Zhen and Bugbee 2020a, 2020b; Zhen et al. 2022) have shown that adding FR on top of PAR increases photosynthetic efficiency. Additional FR in the range 700–750 nm was proposed to have identical photosynthetic efficiency as PAR (400–700 nm) (Zhen and van Iersel 2017; Zhen and Bugbee 2020a, 2020b). These authors proposed that FR in the range 700–750 nm should be included in the definition of PAR. In an extensive study with 14 species, Zhen and Bugbee (2020a) convincingly showed that photosynthetic efficiency of FR was similar as that of PAR when based on incident light, while several of their other studies showed it was similar on the basis of absorbed light (Zhen et al. 2018; Zhen and Bugbee 2020b, Zhen et al. 2022). Some other studies showed no or only small effects of additional FR on photosynthesis (Ji et al. 2019; Kalaitzoglou et al. 2019; Jin et al. 2021; Lazzarin et al. 2025). Despite nonsignificant effects on leaf photosynthesis, Jin et al. (2021) showed that whole‐plant light use efficiency increased under additional FR, as estimated by the ratio of plant dry mass and the cumulative PPFD interception. Therefore, whether additional FR increases photosynthesis rate and dry mass production efficiency is still under debate.

Short‐term effects of the light spectrum on growth and photosynthesis may be different from long‐term effects, due to acclimation of plants to the spectrum. Acclimation to FR has been shown to downregulate photosynthetic capacity, which was related to lower chlorophyll content (Demotes‐Mainard et al. 2016), lower N content and lower mesophyll and stomatal conductance (Wassenaar et al. 2022). Acclimation to FR has also been reported to result in a higher PSII/PSI ratio, decreased Chl a/b ratio, and a higher light‐harvesting complex II (LHCII) concentration per PSII core (Heraut‐Bron et al. 1999; Hogewoning et al. 2012). A question that remains is whether acclimation to FR may affect the strength of the Emerson Enhancement Effect, thus changing the efficiency by which FR powers photosynthesis in combination with PAR. Considering the recent massive interest in FR effects on photosynthetic efficiency, this question is gaining importance.

We aimed to investigate if adding far‐red to PAR light is equally efficient in promoting photosynthesis as adding PAR light, and whether FR should be included in the definition of PAR. Besides quantifying the instantaneous effects of far‐red on photosynthesis, we aimed to study if plants acclimate to far‐red and whether this results in a different photosynthetic response to far‐red. Finally, we aimed to quantify whether whole‐plants light use efficiency is higher in plants grown under a mixture of PAR and FR, compared to growing under PAR only. Lettuce plants were grown in a climate chamber at two levels of PPFD (200 and 400 μmol m^−2^ s^−1^), and at each PPFD level there were also treatments where 25% of PAR or FR was added. In all treatments, the responses of leaf photosynthesis to additional far‐red and PAR were determined. Whole‐plant light use efficiencies were determined based on a growth analysis.

## 2 Materials and Methods

### 2.1 Growth Conditions and Plant Material

Lettuce (*Lactuca sativa* cv. Expertise, Rijk Zwaan, De Lier, the Netherlands) was grown in a climate chamber in three subsequent growing cycles. The chamber was divided in six compartments by white plastic screens to create six treatments. Seeds were sown in watered stonewool plugs in 240‐cell trays (Grodan, Roermond, the Netherlands). During the first 2 days, plugs were placed in darkness at 4°C, and thereafter they were placed for 7 days at 22°C and a photoperiod of 18 h with a PPFD of 142 ± 1.3 µmol m^−2^ s^−1^ provided by red (R) and blue (B) LEDs (88% R and 12% B; GreenPower LED production module DR/B 150 MB, Philips, Eindhoven, the Netherlands). Then, seedlings with two cotyledons were transplanted to stonewool blocks (7 × 7 × 7 cm; Grodan), and grown for 28 days at a density of 148 plants m^−2^. Plants of the outer rows in each compartment were considered border plants and not used for measurements. From transplanting, the photoperiod remained 18 h, temperature was 21.9°C ± 0.1°C, relative humidity was 74.9 ± 0.2% (day) and 78.9 ± 0.0% (night) and CO_2_ concentration ([CO_2_]) was 760.0 ± 4.2 ppm (day) and 730.0 ± 0.9 ppm (night). The nutrient solution (electrical conductivity: 2.3 dS·m^−1^; pH: 5.8) contained 12.9 mM NO_3_^−^, 0.4 mM NH_4_^+^, 1.5 mM H_2_PO_4_^−^, 8.8 mM K^+^, 4.2 mM Ca^2+^, 1.2 mM Mg^2+^, 1.53 mM SO_4_^2−^, 1.5 mM Cl^−^, 0.1 mM HCO_3_^−^, 0.4 mM SiO_3_^2−^, 30.7 µM Fe^3+^, 3.8 µM Mn^2+^, 3.8 µM Zn^2+^, 38.3 µM B, 0.8 µM Cu^2+^ and 0.4 µM Mo, and was applied from the second day after transplanting onwards. The nutrient solution was completely renewed three times a week to keep EC, composition, and pH stable.

### 2.2 Treatments

To study the effects of FR during cultivation on whole‐plant light use efficiency, plants were subjected to six growth light treatments, which were applied for 28 days from transplanting (9 days after sowing) onwards (Table 1). Additionally, in all growth light treatments, instantaneous photosynthesis rates were measured under 14 combinations of RB and FR measurement light. The first three growth light treatments consisted of a reference treatment (200 RB) of 200 µmol m^−2^ s^−1^ of red and blue (RB) light, there was a treatment with additional 50 µmol m^−2^ s^−1^ of RB light (250RB) or 50 μmol m^−2^ s^−1^ of FR light (250FR). The fourth to sixth treatments were similar to the first three treatments, except that all intensities were doubled (400RB, 500RB and 500FR). Photosynthetic photon flux, consisting of 88% Red (R) and 12% Blue (B), was supplied by LED modules (Philips, The Netherlands). The RB light was supplied by GreenPower LED production modules (DR/B 150 MB) in treatments 200RB, 250RB and by GreenPower LED Toplighting modules (DR/B MB) in treatments 400RB, 500RB, and 500FR. Far‐red (FR: 700–800 nm) in treatment 250FR and 500FR was supplied by LEDs (GreenPower LED production modules FR150; Philips). PPFD was measured by a quantum sensor (LI‐250A; Li‐Cor Biosciences, Lincoln, NE, USA) at canopy height and FR PFD by a spectroradiometer (SS‐110; Apogee Instruments, Logan, UT, USA). The spectrum of all treatments is given in Supporting Information S1: Figure S1.

**Table 1:** Overview of six treatments during growth of the plants with their photosynthetic photon flux density (PPFD; 400–700 nm), photon flux density (PFD) of far‐red (FR; 700–800 nm; (82% of these FR photons were in the range 700–750 nm) and total photon flux density (TPFD; 400–800 nm), as well as the total light energy (400–800 nm). Data are the averages of three growing cycles, together with the standard error of means (SEM; *n* = 3). The average value per growing cycle and treatment was determined from 18 measuring spots distributed in the growing area. The energy content of the PPFD (400–700 nm) and FR PFD (700–800 nm) was respectively 0.19 and 0.16 J μmol^−1^.

Growth light treatment	PPFD (µmol m^−2^ s^−1^)	FR PFD (µmol m^−2^ s^−1^)	TPFD (µmol m^−2^ s^−1^)	Light energy (W m^−2^)
200RB	201 ± 1.2	2.5 ± 0.0	204 ± 1.2	38
250RB	247 ± 1.0	0.9 ± 0.0	248 ± 1.0	47
250FR	200 ± 2.0	52.0 ± 5.0	252 ± 7.0	46
400RB	398 ± 11.3	2.2 ± 0.1	400 ± 11.4	76
500RB	500 ± 3.7	1.9 ± 0.1	502 ± 3.7	95
500FR	404 ± 3.4	106.0 ± 5.6	510 ± 9.0	94

The 14 combinations of measurement light were: four levels of additional FR or RB light (0%, 12.5%, 25%, 50% of background PPFD, where background PPFD was 200 or 400 μmol m^−2^ s^−1^ of RB light; for details see Section 2.3.4 Instantaneous effect of far red light on leaf net photosynthesis rate.

### 2.3 Measurements

#### 2.3.1 Plant Growth

Lettuce shoot fresh mass (FM) and dry mass (DM) as well as leaf area (LA) were determined weekly until 28 days after transplanting (DAT) on eight plants per treatment for each of the three growing cycles. Shoot DM was determined after drying by forced air in an oven at 70°C for 2 h, followed by 105°C for 24 h. LA was measured using a leaf area meter (LI‐3100 Area Meter; Li‐Cor Biosciences).

#### 2.3.2 Light and Radiation Use Efficiency of Plant Growth and Absorption of the Canopy

Efficiency of whole‐plant light use was defined as the ratio between final shoot biomass (at 28 DAT) and light integral from transplanting to final harvest. We used several expressions of light and radiation use efficiency: Light use efficiency (LUE_inc_) was calculated as shoot DM divided by incident PPFD (400–700 nm) cumulated over the whole cultivation cycle. Radiation use efficiency of incident radiation (RUE_inc_) was calculated as shoot DM per cumulative incident TPFD (400–800 nm), and radiation use efficiency of absorbed radiation (RUE_abs_) was calculated as shoot DM divided by cumulative absorbed TPFD. Cumulative absorbed TPFD was the sum of daily absorbed TPFD, which was the product of daily incident TPFD and fraction of absorbed radiation.

Based on the law of Lambert–Beer for exponential extinction of radiation in canopies (Monsi 2004), radiation absorption of a canopy (TPFD_abs_, mol m^−2^ d^−1^) can be estimated as (Goudriaan and Van Laar 1993; Marcelis et al. 1998).

(1) $${\mathrm{TPFD}}_{\mathrm{abs}}=\left(1-\rho \right)\cdot {\mathrm{TPFD}}_{\mathrm{inc}}\cdot (1-e^{-k\cdot \mathrm{LAI}})$$

Where *ρ* is the canopy reflection coefficient, TPFD_inc_ is the TPFD incident on the crop (mol m^−2^ d^−1^) that is, the TPFD above the canopy, k is the extinction coefficient of the crop and LAI is leaf area index (m^2^ leaf area per m^2^ of floor area); daily values of LAI were linearly interpolated from weekly measurements. Calculations of TPFD_abs_ were done separately for PAR and FR light and then calculating TPFD_abs_ as the sum of absorbed PAR and FR. Subsequently TPFD_abs_ was cumulated over the 28 days growing period.

*ρ* was estimated using measured leaf optical properties (Figure S2):

(2) $$\rho =\frac{1-\sqrt{1-\sigma }}{1+\sqrt{1-\sigma }}$$

Where *σ* is the wavelength dependent scattering coefficient of the crop. First *σ* was calculated per wavelength as the sum of measured reflection and transmission of individual leaves (for measurements see section on optical properties). Then, *σ* was weighed for the spectral distribution of light per treatment.

*k* was calculated as

(3) $$k=k_{\mathrm{bl}}\cdot \sqrt{1-\sigma }$$

where kbl (0.84) is the extinction coefficient of a crop with spherical leaf angle distribution, when the crop is composed of black leaves.

All calculations above were derived from Goudriaan and van Laar (1993) and as described in Paradiso et al. (2011). LAI was derived from weekly measured leaf area per plant, multiplied by planting density (148 plants m^−2^). Daily values of LAI were estimated by linear interpolation of weekly data.

#### 2.3.3 Photosynthetic Traits Derived From Response Curves

Plants were first dark‐adapted at 22°C air temperature for 20 min before the measurement of maximum and minimum chlorophyll *a* fluorescence (*F*_m_ and *F*_o_, respectively). Maximum photosystem II photochemical efficiency (F_v_/*F*_m_) was determined by a Li‐Cor photosynthesis system together with photosynthesis rate measurement. Measurements on leaves where *F*_v_/*F*_m_ was between 0.7 and 0.8 were accepted for the following data analysis.

Light and CO_2_ response curves of leaf net photosynthesis rate (P_n_) were measured between 21 and 28 DAT in the 2nd and 3rd growing cycles. Per cycle and treatment, four plants were randomly selected, and measurements were conducted on 2 cm^2^ of a fully expanded and unshaded flat leaf, using a Li‐Cor 6800 photosynthesis system (Li‐Cor Biosciences) with the multiphase flash fluorometer (Li‐Cor Part No. 6800‐01 A). The light response curve of P_n_ was conducted at 400 ppm [CO_2_], 22°C air temperature, 75% relative humidity, and 500 µmol s^−1^ flow rate. Before measurements started, leaves were adapted to 1500 µmol m^−2^ s^−1^ PPFD, consisting of 90% R and 10% B for 15 min, subsequently P_n_ was measured at 1500, 1250, 1000, 800, 600, 400, 200, 100, 50, and 0 µmol m^−2^ s^−1^. Thereafter, the CO_2_ response of P_n_ was measured at 1200 µmol m^–2^ s^−1^ PPFD, consisting again of 90% R and 10%. When leaves were adapted to 400 ppm CO_2_ for 15 min, P_n_ was measured at 400, 300, 200, 100, 50, 0, 400, 600, 800, 1000, and 1200 ppm [CO_2_]. At every level of light intensity and CO_2_ concentration, P_n_ was measured when reference H_2_O, reference CO_2_ and stomatal conductance (g_s_) reached steady state (slope < 0.1). Gas exchange rates were corrected in the case of leaves not fully covering the cuvette area by the fraction of the cuvette covered by the leaf. This fraction was determined by ImageJ based on pictures taken from the top of the cuvette with the leaf.

Light response curve data were fitted by a non‐rectangular hyperbola (Ögren and Evans 1993), to obtain α (maximum quantum yield, mol CO_2_ mol^−1^ photons), *P*_max_ (light‐saturated photosynthesis rate, µmol CO_2_ m^−2^ s^−1^), and *R*_d_ (day respiration, µmol CO_2_ m^−2^ s^−1^). CO_2_ response curve data were fitted using the excel tool provided by Sharkey (2016) to obtain VC_max_ (the maximum carboxylation rate of Rubisco), J_1200_ (maximum rate of electron transport at 1200 µmol m^−2^ s^−1^ PPFD and TPU (maximum rate of triose phosphate use).

#### 2.3.4 Instantaneous Effect of Far Red Light on Leaf Net Photosynthesis Rate

Three to four plants per treatment were randomly selected in the 2nd and 3rd growing cycle for quantum yield measurements. A separate compartment in the growing chamber was used, which contained individually dimmable light sources for R (peak at 639 nm), B (peak at 448 nm), and FR (peak at 734 nm; within the range of 700–800 nm 96% of the PFD was in the range the range 700–750 nm; see Supporting Information S1: Figure S3 for spectra). Red and blue were provided by Royal Blue and Red Luxeon K2 LEDs (Lumileds Lighting Company, San Jose, USA). FR light was provided by Philips GreenPower LED FR Research Modules. Gas exchange of the leaves was measured in a clear top chamber of a Li‐Cor 6800 photosynthesis system (Li‐Cor Biosciences). The transmissivity of the clear‐top chamber within the 400–800 nm range was measured by spectroradiometers (SS‐110; Apogee Instruments). The clear‐top chamber was placed underneath the light sources at a fixed position (measuring spot), and a spectroradiometer was placed next to it (reference spot). A linear relationship of light intensities measured in both spots, measured by two spectroradiometers (SS‐110) was established, to identify the photon flux density at the measuring spot during gas exchange measurements. Environmental factors in the Li‐Cor cuvette were: 22°C ± 0.3°C air temperature, 75% ± 5% relative humidity, 1200 ppm [CO_2_], and 500 µmol s^−1^ flow rate. One fully expanded unshaded leaf was selected per plant. To eliminate the possibility of shading, surrounding leaves close to the measuring leaf were removed. The measurement was corrected by fraction of leaf coverage in the chamber in case leaf area was < 6 cm^2^.

Leaves were first adapted to a background RB light of 200 µmol m^−2^ s^−1^ (10% B and 90% R) until P_n_ and g_s_ reached a steady state. Then, gas exchange was measured under four levels of additional FR or RB light (0%, 12.5%, 25%, 50% of background PPFD). Leaves were then acclimated to 400 µmol m^−2^ s^−1^ (RB light) until P_n_ and g_s_ reached steady state and again, FR or RB was added in steps of 12.5%, 25%, 50% of background PPFD. At each light intensity, leaves were given 2 min for P_n_ to stabilize, as in preliminary measurements this had been found to be sufficient to achieve steady‐state P_n_ under these relatively small changes in light intensity. Then, gas exchange data were recorded seven times at a 5 s interval, and resulting data were averaged.

P_n_ was plotted vs. incident and absorbed light intensity. Leaf light absorption was calculated as the integral of the product between leaf absorption rate (see below) and incident light intensity per wavelength (400–800 nm); calculations were done separately per growing cycle.

#### 2.3.5 Pigment Concentrations and Leaf Optical Properties

Three plants per treatment and growing cycle were randomly sampled at harvest (28 DAT). One fully expanded and unshaded leaf was selected per plant, and three leaf discs (total area: 9.42 cm^2^) were collected. After determining their fresh mass, discs were immersed in liquid nitrogen and stored at −80°C. For pigment extraction, leaf discs were pooled in a glass bottle with 3 mL N,N‐dimethyl formamide (DMF, ≥ 99.9% ACS reagent), and kept at −20°C for 14 days, until complete discoloration of leaf discs had occurred. Absorbance of the solution was measured using a spectrophotometer (Varian Instruments, Walnut Creek, CA, United States) at 663.8, 646.8, and 480.0 nm. Concentrations of Chl *a*, Chl *b* and carotenoids were calculated according to Wellburn (1994).

Leaf light transmittance and reflectance were measured by a laboratory‐built system consisting of two integrating spheres as described by Taylor et al. (2019), but with the white LED being replaced by a halogen light source. The absorbance was calculated as incident light minus the sum of transmitted and reflected light.

### 2.4 Statistical Analysis

A randomized complete block design with three blocks (growing cycles) was applied. For each block, a new randomization of the light treatments over the compartments was performed. Homogeneity of variance of the residuals was assumed, and normality of the residuals was shown at *p* = 0.05 with the Shapiro–Wilk test. A two‐way ANOVA analysed plant growth and photosynthesis response curve data; the two factors were background PPFD (200 and 400 µmol m^−2^ s^−1^) and additional light (no addition, 25% added PAR and 25% added FR) during growth. All data were based on three blocks (*n* = 3), except for photosynthesis data, which were based on 2 blocks (*n* = 2). For each TPFD of measurement light, the effects of growth light and measurement light on quantum yield and P_n_ were analysed by a two‐way ANOVA with six growth light treatments and two spectra during photosynthesis measurement (additional R or FR). *F*‐tests were conducted at *p* = 0.05, followed by mean separation according to Fisher's Protected LSD test (*p* = 0.05). As net photosynthesis rate did not show interactive effects of growth light and measurement light, the effects of measurement light were presented as averages over all six growth light treatments across two blocks. Similarly the effects of growth light on photosynthesis and absence or presence of FR in measurement light, were presented as averages over all three additions of RB or FR measurement light of two blocks. All statistical analyses were performed by using Genstat (18th edition, VSN International, Hempstead, UK).

## 3 Results

### 3.1 Instantaneous Effect of Far Red Light on Leaf Net Photosynthesis Rate

**Figure 1:**
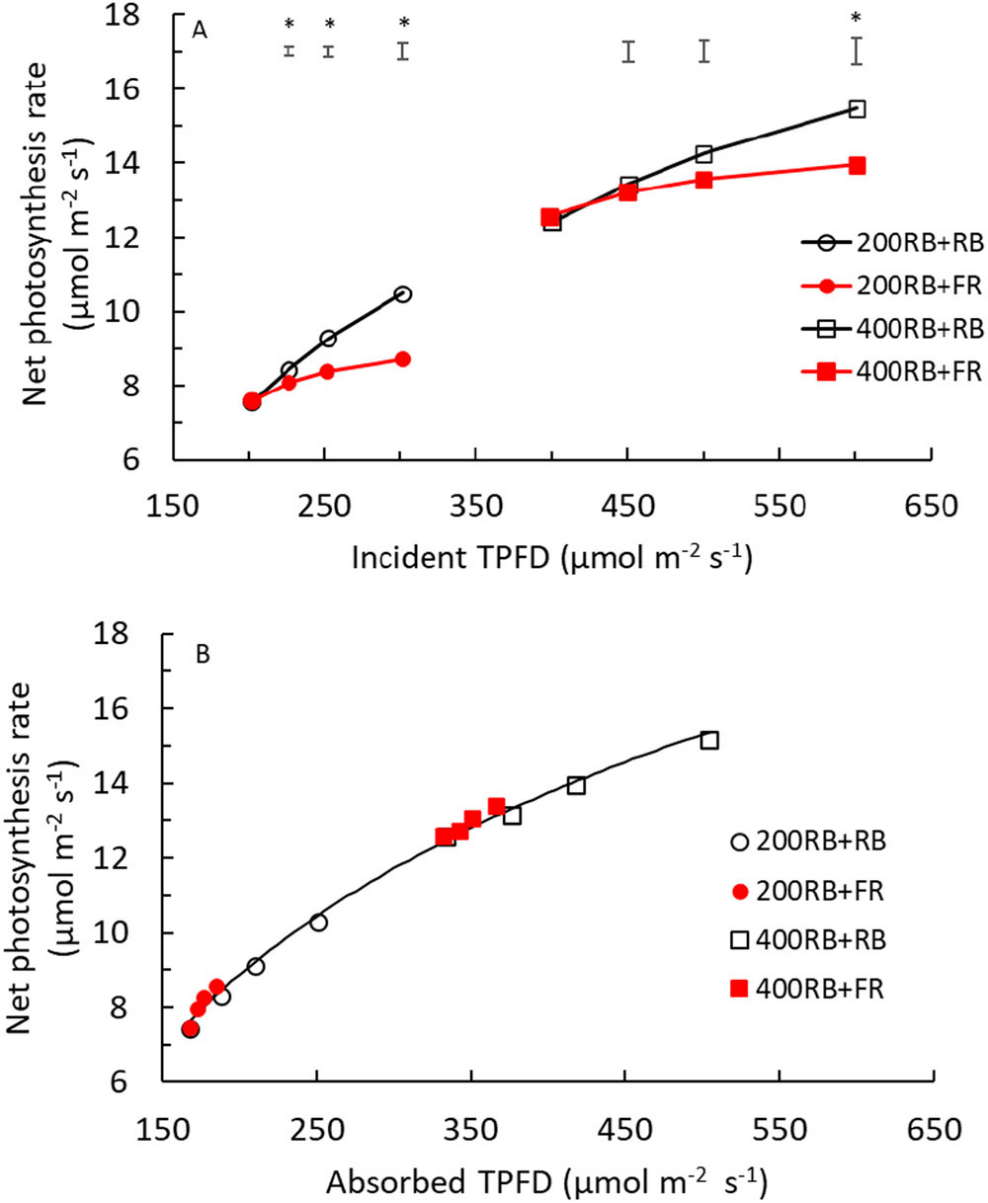
Response curves of leaf net photosynthesis rate (P_n_) to instantaneous total photon flux density (TPFD: 400–800 nm) of measurement light. TPFD refers to incident (A) and absorbed TPFD (B). The measurement light was composed of 200 (circles; 200 RB) or 400 µmol m^−2^ s^−1^ RB light (squares; 400 RB), where 0%, 12.5%, 25% or 50% RB (open symbols) or FR (red closed symbols) was added. Each datapoint represents an average value from all six growth light treatments across two blocks (each symbol was based on 36‐48 plants); there was no significant interaction between measurement light and growth light treatments. 700–800 nm was considered as FR; within this range, 96% of photons were in the range 700–750 nm). SEM was smaller than symbol size (ranging from 0.12 to 0.35 μmol m^−2^ s^−1^); asterisks in panel A indicate that the difference between addition of FR and RB was significant according to an *F*‐test at 5% level. Photosynthesis in the TPFD range 225–300 µmol m^−2^ s^−1^, was significantly different from TPD 200, except for TPFD 225 when the PFD was increased by FR. In the TPFD range 450–600 µmol m^−2^ s^−1^ all photosynthesis rates were significantly different from TPD 400. The line in panel B is the regression line fitted through all data points (*Y* = 7.029ln(X)–28.347; *R* ^2^ = 0.9955; *p* < 0.05). [Color figure can be viewed at wileyonlinelibrary.com]

Adding several intensities of either RB or FR light to a RB background increased P_n_ (Figure 1A). However, P_n_ increased much less strongly when FR was added, compared to when RB was added. At 200 µmol m^−2^ s^−1^ PPFD, the increase in P_n_ due to adding 50% FR was 39% of the increase that was observed when adding 50% PAR, while at 400 µmol m^−2^ s^−1^ PPFD, this increase in P_n_ by adding 50% FR was 45% of the increase due to adding 50% PAR. When only 12.5% FR was added, these increases were a bit higher, at 58% and 64%, respectively. Hence, the effect of adding FR on P_n_ had an efficiency of 39%–64% compared to adding PAR. When comparing to measurements without FR, adding 12.5% FR to background light of 200 or 400 µmol m^−2^ s^−1^ PPFD resulted in, respectively, 6.6% and 5.1% increase of P_n_.

When P_n_ was plotted vs*.* absorbed TPFD, instead of incident TPFD, the curves for light with or without FR were remarkably similar, indicating that absorbed additional FR and absorbed additional PAR (RB) had similar photosynthetic efficiency (Figure 1B).

### 3.2 Acclimation of Photosynthesis Traits and Pigment Concentrations to FR

**Figure 2:**
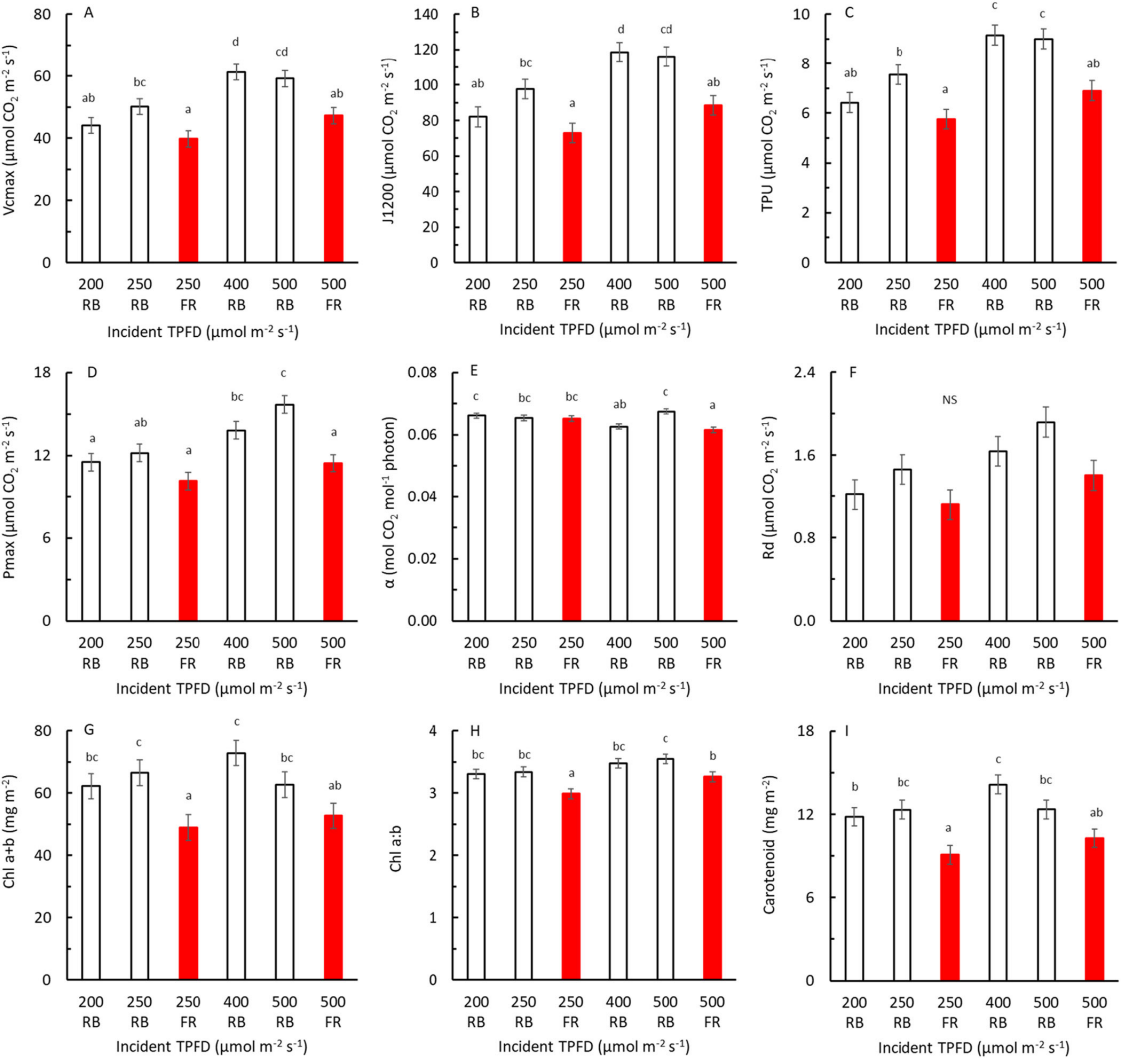
Photosynthetic traits and pigment concentrations in relation to total photon flux density (TPFD) during growth (the growth light). Plants were grown under RB light without FR (black symbols) or were grown under RB light with 25% FR (red symbols), at four TPFD levels (legends refer to grow light treatments as detailed in Table 1). The Measurements of the photosynthetic traits were conducted under red (90%) and blue (10%) light. The maximum carboxylation rate of Rubisco (VC_max_, A), rate of electron transport at 1200 µmol m^−2^ s^−1^ PPFD (J_1200_, B), maximum rate of triose phosphate use (TPU, C), light‐saturated net photosynthesis rate (*P*_max_, D), maximum quantum yield (*α*, E), day respiration (*R*_d_, F), Chl a + b (G), Chl a:b (H), and carotenoid content (I) are shown. Symbols show means ± SEM and were based on two blocks each with three to four replicate plants. NS means no significant effects at 5% level. [Color figure can be viewed at wileyonlinelibrary.com]

Acclimation to FR light added to PAR or partly substituting PAR during growth of the plants reduced photosynthetic capacity, respiration rate, content of chlorophyll a + b, the chlorophyll a:b ratio, and total carotenoid content, at both background light intensities (Figure 2). The reduction in photosynthetic capacity was apparent from reduced VC_max_, J_1200_, TPU, and *P*_max_ when FR was added to PAR or when FR partly substituted PAR during plant growth (Figure 2a–d). Also, acclimation to increased PAR light intensity generally caused an increase in photosynthetic capacity; the traits VC_max_, J_1200_, TPU, and *P*_max_, increased when PPFD increased from 200 to 400 μmol m^−2^ s^−1^ (Figure 2a–d). The maximum quantum yield (*α*) was hardly affected by spectrum or TPFD of the growth light (Figure 2e).

**Figure 3:**
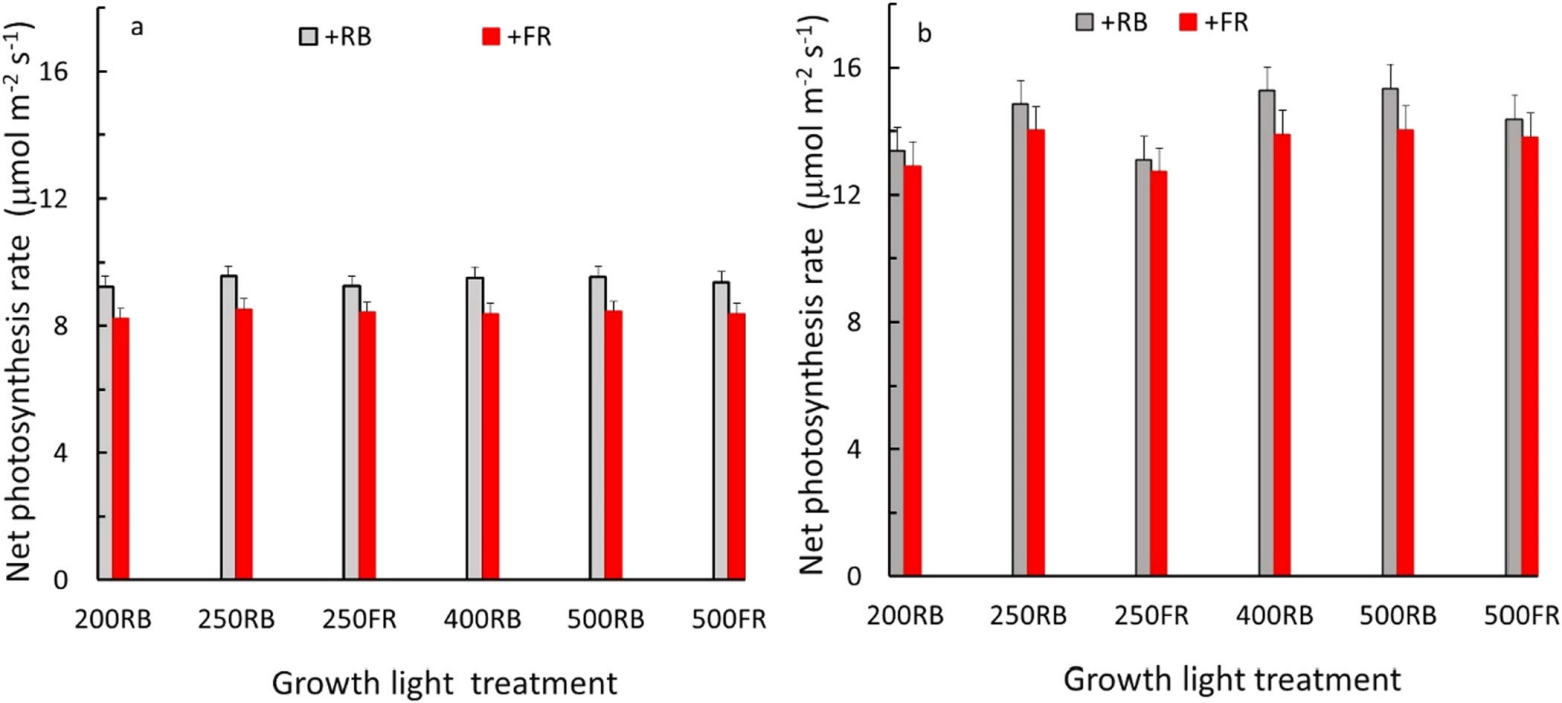
Effects of growth light conditions on leaf net photosynthesis (P_n_). Growth light conditions were 200 or 400 µmol m^−2^ s^−1^ RB background light and there were also treatments with 25% of PAR or FR added (see Table 1). (a and b) differ in measurement light: The measurement light was composed of 200 (a) or 400 µmol m^−2^ s^−1^ RB light (b), where 12.5%, 25%, or 50% RB (grey bars) or FR (red bars) was added. Each bar represents the average value from all three additions of RB or FR measurement light of two blocks (each bar was based on 18–24 plants, which is 3–4 plants times 3 measurement lights, times 2 blocks). Error bars represent SEM; Effects of growth light treatments and the interaction with measurement light were not significant, while effect of measurement light (RB or FR) was significant at 5% level. [Color figure can be viewed at wileyonlinelibrary.com]

The growth light treatments (different levels of RB and FR intensity) had no significant effect on P_n_ when measured under RB nor when measured under a mixture of RB and FR (Figure 3). Under all growth light conditions, P_n_ measured under light with FR was lower than under light with only RB (Figure 3).

### 3.3 Effects of FR on Plant Growth and Light Use Efficiency

**Figure 4:**
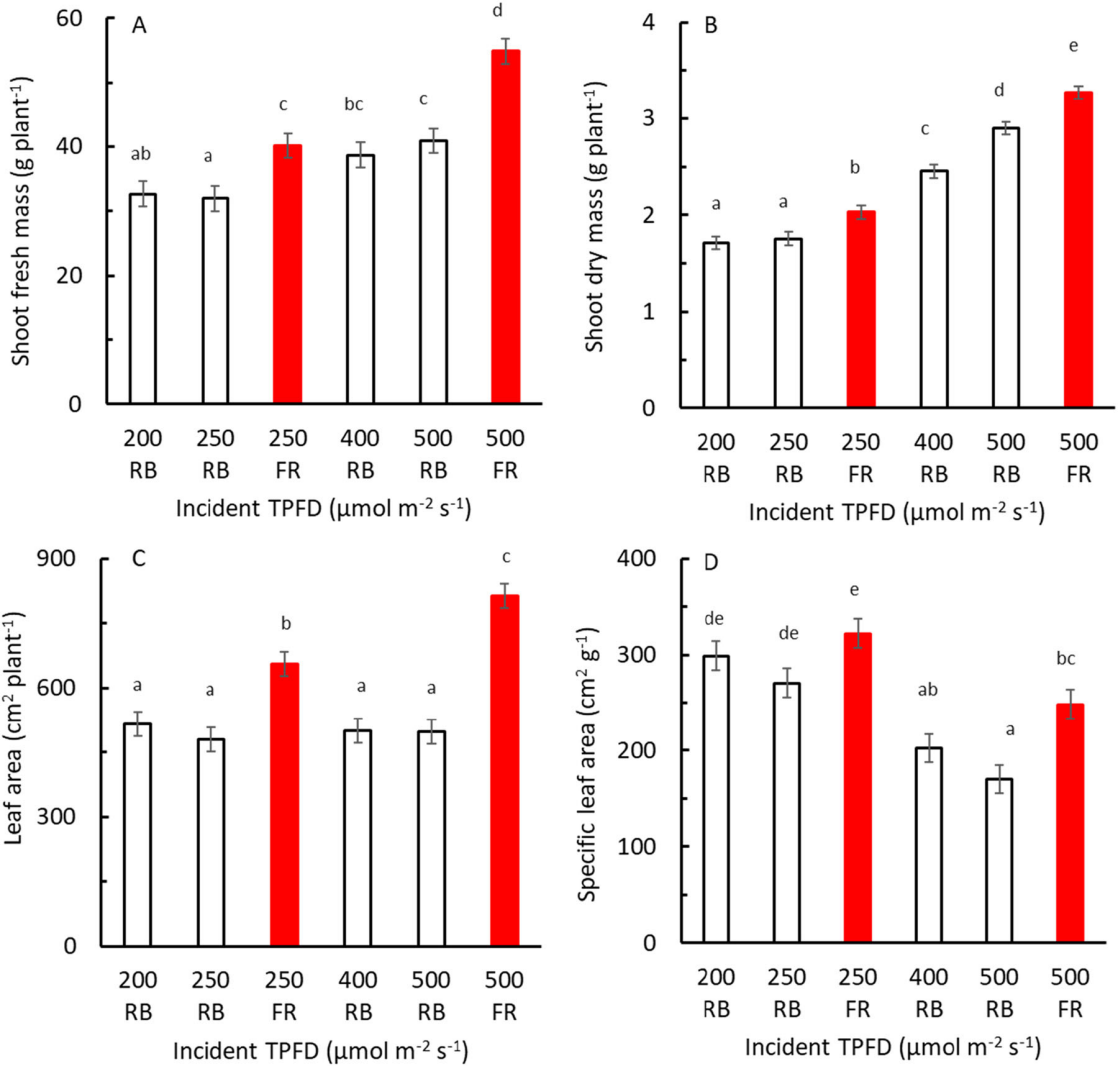
Lettuce shoot fresh mass (A), shoot dry mass (B), leaf area (C), and specific leaf area (D) at harvest (28 DAT) in response to total photon flux density (TPFD) during growth (growth light). Plants were grown under RB light without FR (black symbols) or were grown under RB light with 25% FR (red symbols). Symbols show means ± SEM and were based on three blocks each with eight replicate plants. [Color figure can be viewed at wileyonlinelibrary.com]

When the growth light contained FR, the fresh and dry mass as well as leaf area and specific leaf area of the shoots was higher than when growth light did not contain FR when comparing at equal TPFD (Figure 4). These effects were relatively larger for fresh mass than for dry mass.

In the first part of the experiment when LAI was still low, adding FR or partly replacing PAR by FR resulted in larger light as well as radiation interception, due to an increased leaf area index (which has a strong effect on interception as long as LAI is low) (Supporting Information S1: Figure S4). However at the end of the experiment, when the canopy was fully closed (high leaf area index), radiation interception was largest for treatments without FR (due to the larger absorption of PAR compared to FR), when comparing treatment effects at the same TPFD (Supporting Information S1: Figure S4).

**Figure 5:**
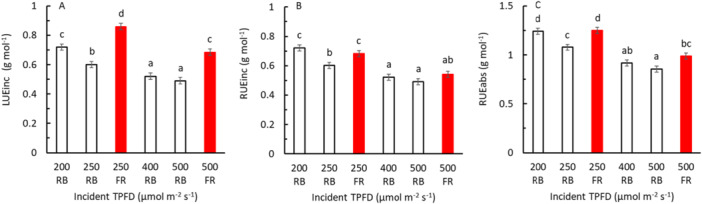
Light and radiation use efficiency of lettuce plants in response to total photon flux density (TPFD) during growth (growth light). Plants were grown for 28 days under RB light without FR (black symbols) or were grown under RB light with 2 5% FR (red symbols). (A) light use efficiency (LUE_inc_) refers to the shoot dry mass per cumulative incident PPFD (400–700 nm). (B) radiation use efficiency (RUE_inc_) refers to the shoot dry mass per cumulative incident TPFD (400–800 nm). (C) Radiation use efficiency (RUE_abs_) refers to the shoot dry mass per cumulative absorbed TPFD (400–800 nm). Symbols show means ± standard error of means (when larger than symbol size) and were based on three blocks each with eight replicate plants. [Color figure can be viewed at wileyonlinelibrary.com]

Here, LUE_inc_ is defined as the shoot dry mass per unit cumulative incident PPFD (400–700 nm), while RUE_inc_ and RUE_abs_ are calculated per unit of cumulative incident or absorbed TPFD (400–800 nm), respectively. Adding FR distinctly increased LUE_inc_, regardless whether the comparison was made at the same incident PPFD or at the same TPFD (Figure 5a). When comparing at the same TPFD, RUE_inc_ as well as RUE_abs_ were higher when the growth light contained FR compared to growth light without FR, but this effect was not statistically significant for RUE_inc_ at a TPFD of 500 μmol m^−2^ s^−1^ (Figure 5b,c).

## 4 Discussion

There is compelling evidence that photons from 700 to 750 nm should be considered as photosynthetically active (Zhen and Bugbee 2020a, 2020b; Zhen et al. 2021; Zhen and Bugbee 2021; Zhen et al. 2022) when added to PAR. These authors proposed to extend the definition of photosynthetically active radiation to include photons from 400 to 750 nm with the acronym ePAR (extended PAR), which would be an improved metric that better predicts photosynthesis under a range of light sources (including sunlight) than PAR. In the present study, FR effects on biomass production and light use efficiency of lettuce plants grown under LED light were explored, and whether FR is identically efficient in improving leaf net photosynthesis rate as PAR and whether this depends on acclimation of plants to FR. We found that FR is definitely photosynthetically active, but not to the same extent as PAR. However, when the photosynthetic efficiency was expressed per mole of absorbed instead of incident photons, the efficiency of FR was rather similar to that of PAR (when PAR and FR were supplied in combination). So, when characterizing the light environment it is not sufficient to limit this to PAR, but also FR needs to be considered; for instance by also providing ePAR.

### 4.1 FR Increased Leaf Photosynthesis Rate Less Strongly Than PAR

It was reported before (Zhen and van Iersel 2017; Zou et al. 2019) that adding FR to PAR can increase leaf net photosynthesis rate (P_n_) per unit leaf area. Similarly, we found that additional FR increased P_n_ at 200 and 400 µmol m^−2^ s^−1^ background PPFD (Figure 1). However, the P_n_ increments by additional FR were reduced, as the fraction of FR added increased. This is in line with Zhen and van Iersel (2017), who added six different FR photon fluxes from 0 to 90 µmol m^−2^ s^−1^ to 200 µmol m^−2^ s^−1^ RB in lettuce and found that P_n_ increased asymptotically with increasing FR photon flux. Since adding FR can balance the excitation levels of PSI and PSII, the incremental effect on P_n_ by additional FR is associated with PAR. The FR effect on P_n_ saturates when FR starts to overexcite PSI.

In our study with lettuce leaves, we found FR to be less photosynthetically active than PAR; photosynthesis being about 39%–64% as efficient (Figure 1). This is in line with Zhen et al. (2022) measuring leaf photosynthesis when filtering FR from solar light. However, Zhen and Bugbee (2020a) showed that FR had similar whole‐canopy photosynthetic efficiency as PAR in 14 species (including lettuce, while for lettuce this was also shown at leaf level). On the other hand, Lazzarin et al. (2025) found only small effects of FR on leaf photosynthesis in tomato. In our study, the efficiency of leaf photosynthesis per mole of absorbed light was rather similar for RB and FR light, which is line with Zhen et al. (2018) and Zhen and Bugbee (2022), while Zhen and Bugbee (2020b) showed similar efficiency of canopy photosynthesis per mole of absorbed light. This suggests that the difference in efficiency between FR and RB light is due to lower absorption of FR light. When considering a dense canopy, the difference in absorption of FR versus RB will be smaller than for a single leaf. Assuming that the fraction of absorbed light by a single leaf would be 88% for RB and 12% for FR (as measured in the present study; Supporting Information S1: Figure S2), the estimated fraction of radiation absorbed by the canopy with a leaf area index of 3 would be 88% for RB and 30% for FR (based on Equations (1), (2), (3)).

The FR spectra used for photosynthesis measurements in the present study and in that of Zhen and Bugbee (2020a) had the same peak, at about 735 nm (Supporting Information S1: Figure S5). Hence, a difference in the spectrum of FR cannot explain different responses of P_n_ to FR between these studies. Effects of FR on photosynthesis may depend on the PAR level, being stronger at lower PAR levels (Lazzarin et al. 2025). However, considering the range of PAR levels in our study (200–400 μmol m^−2 ^s^−1^ PAR) and the studies of Zhen and Bugbee, this neither seems a likely explanation for observed differences. Furthermore, the spectrum of the background light or the cultivar may play a role.

Our data clearly show that FR is photosynthetically active when supplied in combination with other wavelengths (Figure 1). Therefore, we agree that for studies on photosynthesis it is not sufficient to only report the intensity of PAR light in the range of 400 to 700 nm. Also, the intensity of FR (700 to 750 nm) should be reported. The term ePAR might be a useful addition when describing experimental conditions, however, we propose to present conventional PAR (400–700 nm) as well as FR may have quite a different effect on photosynthesis than PAR. Ideally, one would weigh the efficiency of the different wavelengths in a new definition of PAR, but at present it does not seem feasible to apply a weighed ePAR that would hold under a wide range of conditions and crops. Additionally, it was recently demonstrated that UV‐A1 radiation (350–400 nm) caused a positive (albeit in some cases very small) photosynthetic quantum yield in a range of species (Sun et al. 2025), suggesting that a future re‐definition of PAR could include the waveband range 350–750 nm.

Furthermore, FR might have even more prominent effects on many other plant processes regulated via phytochromes, making it very important to know the far‐red intensity (e.g., Demotes‐Mainard et al. 2016; Lazzarin et al. 2021). For phytochrome responses, wavelengths between 750 nm to almost 800 nm can be also relevant (Christie and Zurbriggen 2020). Therefore, in this paper we considered photons ranging from 700 to 800 nm as FR. The light source used in the photosynthesis measurements hardly contained photons above 750 nm (only 4% of FR photons). However, in the growth light, 18% of the photons were in the range 750–880 nm.

### 4.2 Acclimation to FR Did Not Affect Photosynthetic Quantum Yield

Leaves tune their photosynthetic apparatus to the spectrum of their growth environment. Our results showed that when lettuce plants are grown for a prolonged period under light where part of the PAR is replaced by FR, leaves acclimate through reduced VC_max_, TPU, J_1200_, P_max_, and R_d_, as well as chlorophyll and carotenoid contents (Figure 2). Similarly, a decreased photosynthetic capacity was found in tomato (Ji et al. 2019; Kalaitzoglou et al. 2019), cucumber (Hogewoning et al. 2012), and lettuce (Zou et al. 2019; Kong and Nemali 2021) when part of PAR was replaced by FR. Also, mesophyll and stomatal conductance may be reduced when plants are grown with additional FR (Kalaitzoglou et al. 2019; Wassenaar et al. 2022). Despite the abovementioned acclimation of leaves when grown for a prolonged period with or without additional FR, the instantaneous photosynthetic response to additional FR in the measuring light was not affected by FR being present or absent in the growing light (Figure 3).

### 4.3 FR Improves Light Use Efficiency

Several researchers studied the effects of FR on lettuce grown in vertical farming conditions, where plants are grown under artificial light and where all growth conditions are usually kept constant (Meng and Runkle 2019; Zou et al. 2019, 2021; Zhen and Bugbee 2020b; Jin et al. 2021; Legendre and van Iersel 2021; Van de Velde et al. 2023). All these studies (except for a study of Kusuma and Bugbee (2023) who found the effects to depend on the light level in young lettuce plants) showed that adding FR on top of PAR improved whole‐plant light use efficiency based on incident PPFD, which was at least partly due to increased leaf expansion leading to enhanced light interception by the canopy, which is in line with our findings (Figure 5a). Consequently, it was recently proposed to pursue a more dynamic approach to environmental control in vertical farming systems, which could include targeted FR application during early crop development to increase light interception (Kaiser et al. 2024).

So far, most studies on FR compared treatments with the same PPFD rather than the same TPFD, whereas in our study we did both. We show that LUE also increased when compared at the same TPFD, that is, when PAR is partly substituted by FR (Figure 5a). Jin et al. (2021) studied the effects of FR at three planting densities (23, 37 and 51 plants m^−2^). They found that the positive effects of additional FR on LUE were smaller the higher the planting density was. This can be explained by the fact that at high planting density, the leaf area index is relatively high and therefore, the stimulation of leaf area expansion has limited effects on radiation interception. At low planting density, on the other hand, FR effects on leaf area expansion and radiation interception are dominant. We performed this experiment at a high planting density (148 plants m^−2^), as we aimed to focus on aspects of photosynthesis. At the end of the experiment, the minimum leaf area index was 7.6 in the absence of FR, which suggests that differences in the fraction of absorbed radiation due to leaf area differences between treatments were relatively small. Considering the findings of Jin et al. (2021) that effects of FR on LUE gets smaller at higher plant density and that RUE decreases with increasing TPFD, it may be not so surprising that in this study with plants grown at high plant density, no significant effect was found on RUE_inc_ when TPFD was increased by adding FR. However, when plants were compared at the same TPFD, partly replacing PAR by FR, we did find increased RUE_inc_ (Figure 5b).

Finally, we showed that RUE_abs_ was increased when PAR was substituted by FR. This was also evident from the dry matter increase during the last week of the experiment, when the canopy was fully closed: substituting 25% of PAR by FR enhanced the dry matter increase during the last week by about 10% (though not statistically significant; Supporting Information S1: Figure S6).

Considering that additional FR improved leaf photosynthesis less strongly than additional PAR, this positive effect of FR on RUE_abs_ cannot be attributed to leaf photosynthesis. Maybe changes in plant architecture together with the different extinction of PAR and FR light, caused canopy photosynthesis to increase. Another reason that absorbed photons (400–800 nm) are more efficiently used when the spectrum contains FR could be that the construction and maintenance costs of dry matter are reduced under FR light. The construction and maintenance costs depend on the composition of the dry matter (De Vries et al. 1974; Amthor 1989). FR light leads to higher carbohydrate content in lettuce (Van Brenk et al. 2024), which has much lower construction costs than most other compounds of plant dry mass, while phenolics content tended to decrease at high levels of FR (Van de Velde et al. 2023). Further research including detailed measurements of light distribution and leaf photosynthesis within the canopy grown under light with and without FR, as well as measuring the chemical composition of the plant dry matter might help to further understand the increased efficiency at which absorbed photons are used for growth.

## 5 Conclusions

Our results clearly showed that FR is photosynthetically active when supplied in combination with other wavelengths. However, in light mixtures of FR with PAR, leaf photosynthetic efficiency under FR is about 39%–64% of that of PAR. This difference is mainly due to lower absorption of FR photons, as on basis of absorbed photons, photosynthetic efficiency of FR and PAR are rather similar. Therefore, for studies on photosynthesis it is not sufficient to only report the intensity of PAR light in the range of 400 to 700 nm.

When grown for a prolonged period with or without FR, acclimation occurs as shown by a reduction of a number of photosynthetic parameters (VC_max_, TPU, J_1200_, P_max_, and R_d_, as well as content of chlorophylls and carotenoids) when grown with FR compared to plants gown without FR. Despite this acclimation, the instantaneous leaf photosynthetic response to additional FR in the measuring light was not affected by the presence or absence of FR in the growing light.

Even though absorbed PAR and FR photons (when supplied in combination) appeared to have equal efficiency for leaf photosynthesis, FR increased the efficiency with which absorbed photons were used for dry matter growth.

## Conflicts of Interest

The authors declare no conflicts of interest.

## Supporting information

Supmat.

## Data Availability

The data that support the findings of this study are available from the corresponding author upon reasonable request.
