# Supplementary material for: Incident Far‐Red Photons Drive Leaf Photosynthesis Less Efficiently Than PAR Light, but Are More Effective in Promoting Growth
Source: Plant Cell Environ. 2025 Sep 16;48(12):9025–35. doi: 10.1111/pce.70193 (PMC12586904; doi:10.1111/pce.70193)
Supplement: Supplementary file 1 — Supmat. [file PCE-48-9025-s001.docx]

$TPFD_{abs}=\left( 1-\rho\right)\cdot TPFD_{inc}\cdot(1-e^{-k\cdot LAI})$ $\rho=\frac{1-\sqrt{1-\sigma}}{1+\sqrt{1-\sigma}}$ $k=k_{bl}\cdot\sqrt{1-\sigma}$

**Supplementary data**

**
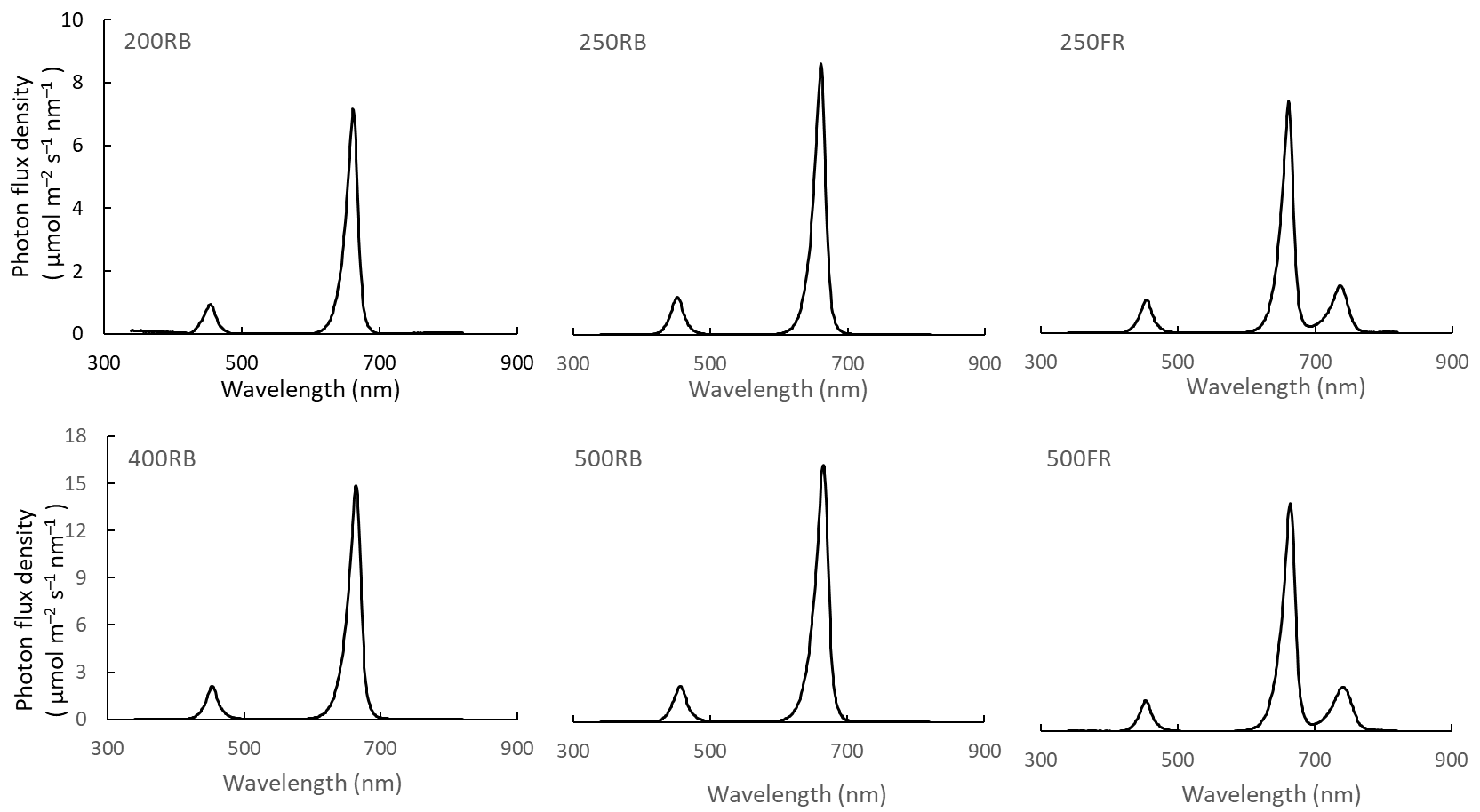
**

Fig. S1. Spectral distribution of growing light in all treatments (200RB, 250RB, 250FR, 400RB, 500RB, 500FR). The spectra were measured at canopy level, and are the average of 18 locations per treatment on a horizontal plane within a given growing compartment. Blue LEDs peaked at 454 nm, red at 664 nm and FR at 739 nm (within the range 700-800 nm 82 % of photons were in the range 700-750 nm).

Fig. S2. The effect of growth light treatments on leaf light absorptance. Data were the means of crop cycles 2 and 3, where each cycle was based on 3 to 5 replicate plants.


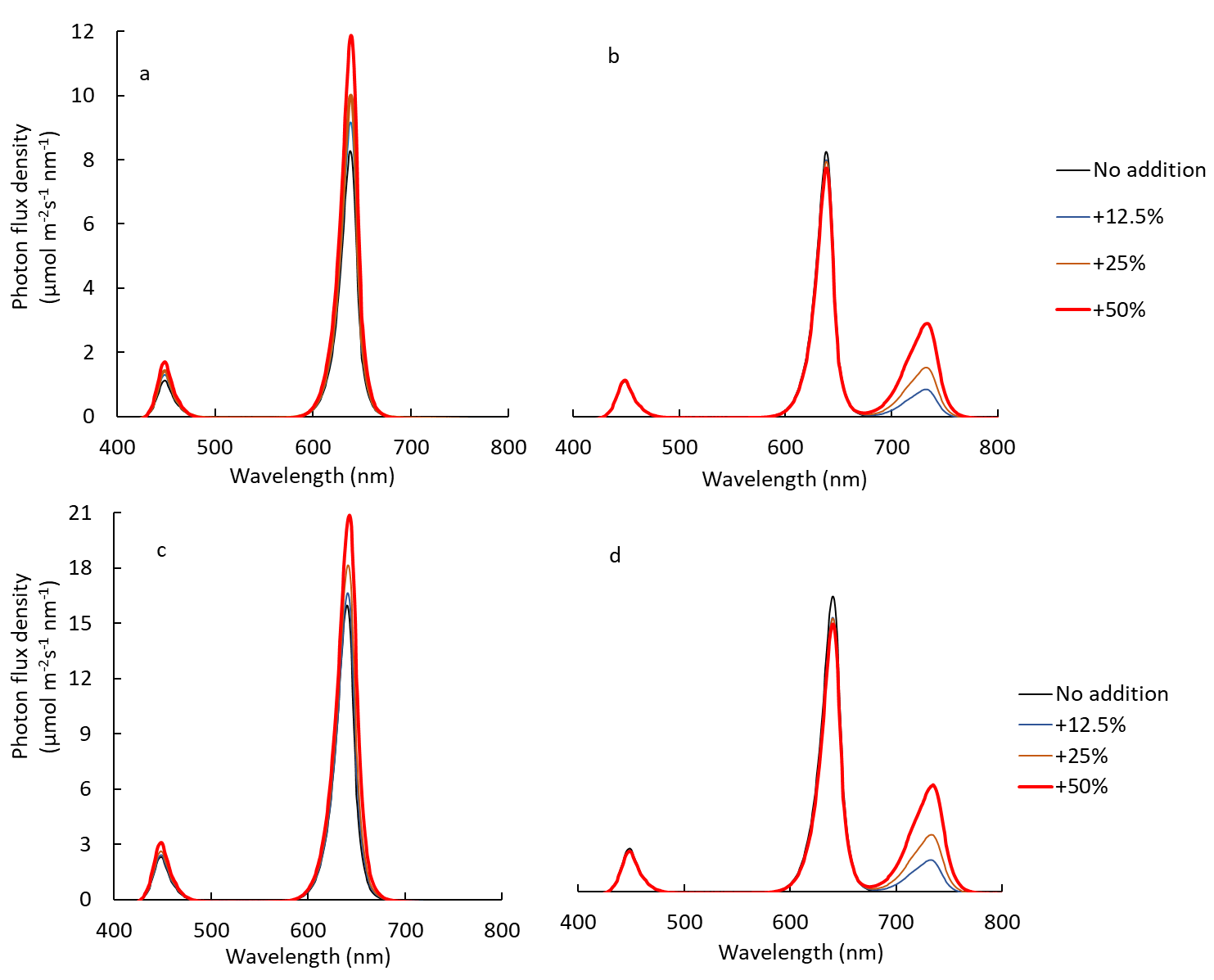


Fig. S3. Spectral distribution of measuring light for photosynthesis. The spectral distribution of adding four levels of RB light (a, c) or FR (b, d) to 200RB (a, b) or 400 RB (c, d) background light. Data are the average of cycles 2 and 3. Legend numbers indicate the intensity of additional RB or FR light as percentage of the base lighting. Blue LEDs peaked at 448 nm, red at 638 nm and FR at 734 nm (96 % of photons in the range 700-800 nm were in the range 700-750 nm).


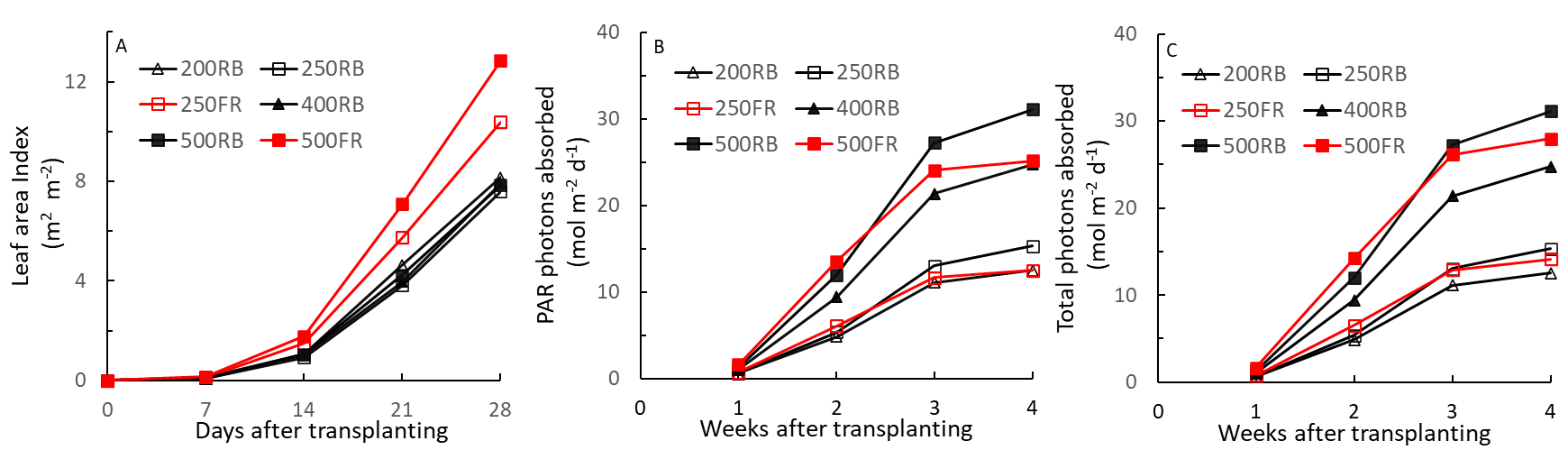


Fig. S4. Time courses of leaf area index (A), canopy absorption of absorbed PAR photons (B) and total photons. For legends see Table 1. Data were based on three blocks each with eight replicate plants.


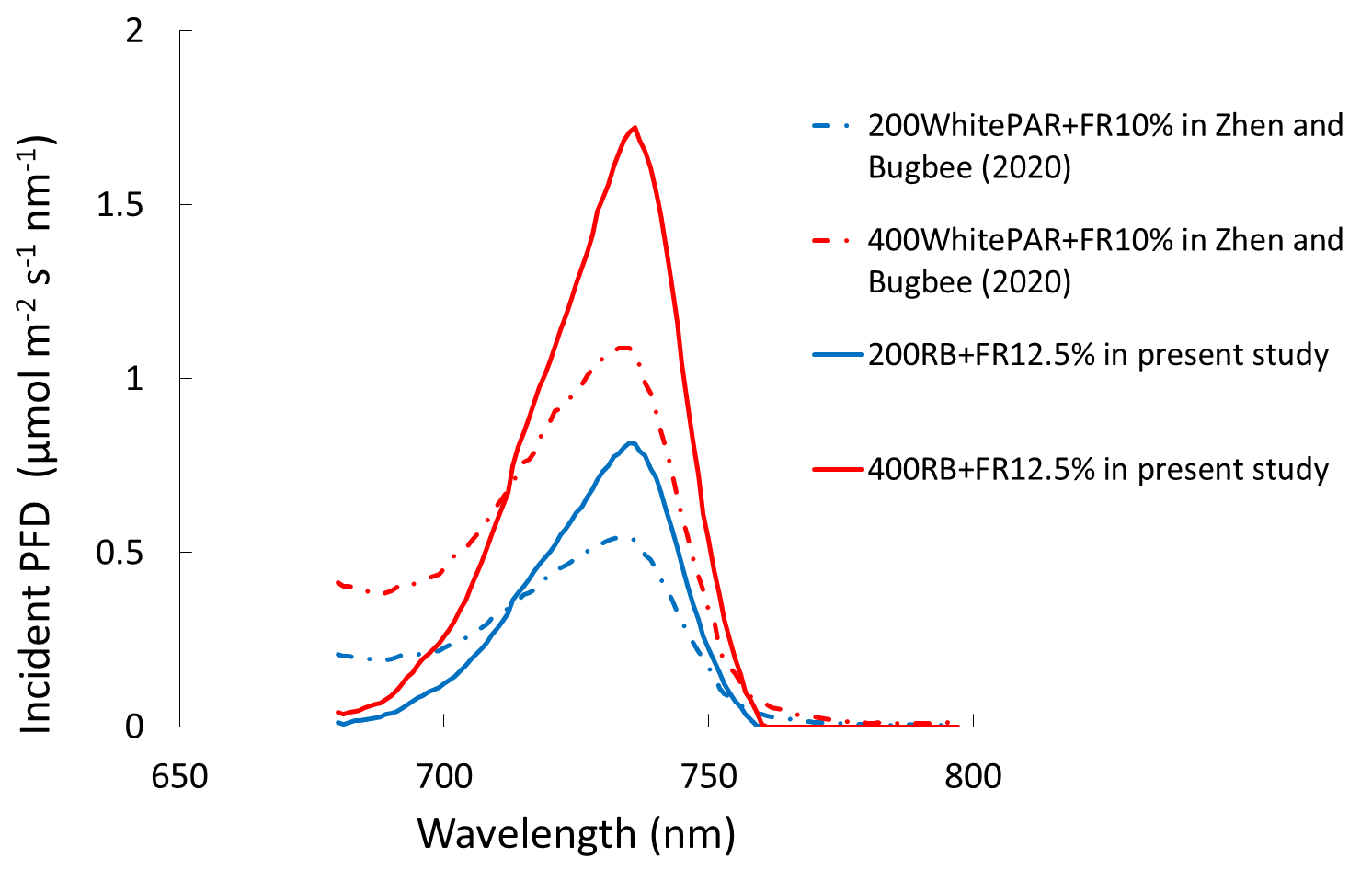


Fig. S5. Comparison of spectral distribution of FR used in our photosynthesis measurements and in Zhen and Bugbee (2020a).


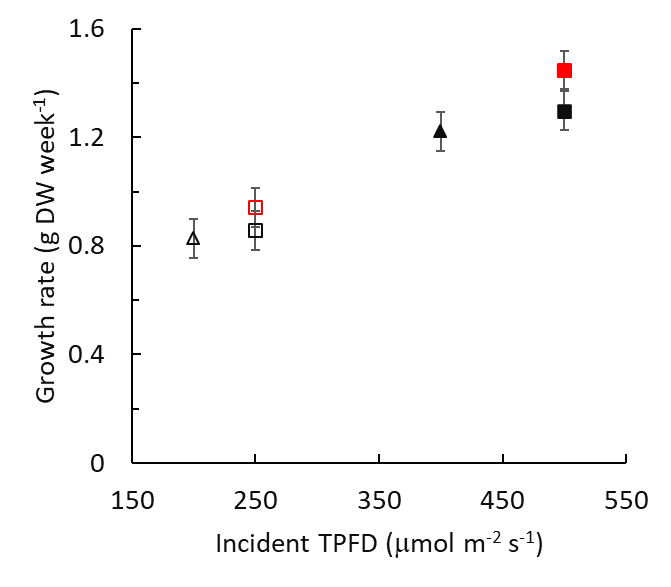


Fig. S6. Growth rate per plant during the last week of cultivation (21-28 DAT). Plants were grown for 28 days under RB light without FR (black symbols) or were grown under RB light with 25 % FR (red symbols). Symbols show means ± standard error of means and were based on three blocks, each with eight replicate plants.
